# Einsamkeit: Ein Begriff für viele Wirklichkeiten

**DOI:** 10.1007/s00103-024-03947-w

**Published:** 2024-09-25

**Authors:** Joseph Kuhn, Martin Härter, Peter Brieger, Steffi G. Riedel-Heller

**Affiliations:** 1 Bayerisches Landesamt für Gesundheit und Lebensmittelsicherheit, Veterinärstr. 2, 85764 Oberschleißheim, Deutschland; 2 Zentrum für Psychosoziale Medizin, Institut und Poliklinik für Medizinische Psychologie und Institut für Psychotherapie (IfP), Universitätsklinikum Hamburg-Eppendorf, Martinistr. 52, 20246 Hamburg, Deutschland; 3 Kbo-Isar-Amper-Klinikum, Akademisches Lehrkrankenhaus der LMU, Vockerstr. 72, 85540 Haar, Deutschland; 4 Institut für Sozialmedizin, Arbeitsmedizin und Public Health, Universität Leipzig, Philipp-Rosenthal-Str. 55, 04103 Leipzig, Deutschland

Im 19. Jahrhundert gab es zum Zusammenhang von Einsamkeit und Gesundheit noch nichts zu sagen. In Meyers Konversationslexikon von 1886 findet sich als Eintrag zum Stichwort Einsamkeit nur dieser Hinweis: „Einsamkeit (Ensomheden), eine vom norwegischen Kapitän E.H. Johannesen aus Tromsö benannte einzelne Insel, welche er am 16. Aug. 1878 im westsibirischen Eismeer nordwestlich von Kap Taimyr … entdeckte. Sie ist 18,5 km lang, hat 202 qkm Flächeninhalt und steigt auf der Westseite bis zu 30 m Höhe empor, während die Ostseite flach und von Treibholz bedeckt ist. Das Innere der Insel war frei von Schnee, aber ohne alle Vegetation.“

In der älteren Literatur wird Einsamkeit eher mit dem gewollten Rückzug aus der Welt, paradigmatisch dem kontemplativen Einsiedler, in Verbindung gebracht. Auch bei Nietzsche, der das Leiden an der Einsamkeit kannte und beschrieb, ist die Einsamkeit zugleich der Ort der Distanzierung von den Niederungen der Welt, ein Ort des Zu-sich-Findens. Diese Seite hat Einsamkeit auch heute noch – nicht wenige Menschen wollen z. B. dem Stress der modernen Zivilisation durch Wanderungen in einsamen Regionen oder durch Angebote wie „Kloster auf Zeit“ entgehen. Aber häufiger geht es inzwischen um die ungewollten und negativen Konsequenzen, um Einsamkeit als Befindlichkeitsstörung und als Gesundheitsrisiko.

Einsamkeit ist als Gesundheits- und wissenschaftliches Thema ein junges Gebiet, allerdings eines mit Konjunktur. Die Literaturdatenbank PubMed verzeichnet bis in die 1970er-Jahre jährlich kaum eine Handvoll von Artikeln, die unter „loneliness“ verschlagwortet sind, bis zur Jahrtausendwende waren es weniger als 100 Arbeiten jährlich, in den letzten 3 Jahren jeweils deutlich mehr als 2000 Publikationen.

In Deutschland gaben vor der Coronakrise im Sozio-ökonomischen Panel (SOEP) gut 2 % der Erwachsenen an, sich häufig oder sehr häufig einsam zu fühlen. 2021, auf dem Höhepunkt der Coronakrise, waren es etwa 5‑mal so viele Personen. Die NAKO-Gesundheitsstudie zeigte, dass fast jeder dritte Deutsche im Mai 2020 sich als einsam bezeichnete. Je nach Erhebungsmethode fallen die Daten etwas unterschiedlich aus, aber überall fiel der Anstieg bei Frauen und bei jungen Erwachsenen besonders stark aus. Der Maximalwert der Betroffenenzahlen dürfte mit dem Abklingen der Coronakrise überwunden sein, die Zahlen normalisieren sich wieder und mit etwas Glück halten sich auch die gesundheitlichen Folgen dieser Krisenreaktion in Grenzen. Dessen ungeachtet bestehen aber keine Zweifel mehr daran, dass Einsamkeit ein ernst zu nehmendes Gesundheitsrisiko ist.

Einsamkeit stellt zum einen selbst eine Einschränkung des Wohlbefindens dar, zum anderen kann sie zu Befindlichkeitsstörungen wie Schlaflosigkeit, Niedergeschlagenheit oder Ängsten führen, aber auch zu manifesten psychischen Störungen wie Depressionen oder zu körperlichen Erkrankungen wie Herz-Kreislauf-Erkrankungen oder Diabetes. Einsamkeit kann zu vermehrtem Konsum von Alkohol oder Tabak führen und sie erhöht das Sterblichkeitsrisiko. Mit anderen Worten: Es gibt kaum eine Erkrankung, deren Auftretensrisiko im Zusammenhang mit Einsamkeit nicht erhöht ist. Umgekehrt können natürlich auch schwere Krankheiten einsam machen. Das gilt nicht nur für immer noch schambehaftete Krankheiten wie die psychischen Störungen oder schwere chronische Erkrankungen wie Krebs, sondern auch für Krankheiten, die die Mobilität einschränken oder Ängste auslösen, nicht belastbar zu sein, z. B. bei Patient:innen nach Herzinfarkt.

Im Vereinigten Königreich ist 2018 eine ministerielle Zuständigkeit für Einsamkeit im Sportministerium geschaffen worden, 2021 wurde in Japan ein Minister für Einsamkeit ernannt und auch die Koalitionsvereinbarung der deutschen Regierung sieht Einsamkeit als eines der Schwerpunktthemen für den Nationalen Präventionsplan vor, im Dezember 2023 wurde eine „Strategie der Bundesregierung gegen Einsamkeit“ vorgelegt. Im Mai 2023 hat der US Surgeon General Einsamkeit dann als „a public health problem on the scale of smoking“ bewertet – also auf der Ebene des bislang größten verhaltensbedingten Gesundheitsrisikos angesiedelt.

Ist somit die Einsamkeit einerseits auf dem Gipfel der gesundheitspolitischen Wahrnehmung angekommen, ist sie andererseits ein weitgehend unbekanntes Wesen, ein schillerndes dazu. Die Wissenschaft ist sich einig, dass Einsamkeit als subjektives Empfinden abzugrenzen ist von der sozialen Isolation als einem objektiven Sachverhalt. Ebenso besteht Konsens darüber, dass man auch in Gesellschaft einsam sein kann. Menschen, die Mobbing ausgesetzt sind, sind beispielsweise einsam, weil z. B. negative soziale Beziehungen sie gezielt einsam machen. Junge Menschen, die viele „Freunde“ in sozialen Foren haben, können sich trotzdem einsam fühlen, ebenso wie man sich in Partnerschaften unverstanden und einsam fühlen kann. Des Weiteren hat sich mit der erwähnten Zunahme der Studien zu Einsamkeit und Gesundheit eine große Menge Daten dazu ergeben, die belegen, mit welchen Krankheiten Einsamkeit zusammenhängt, welche Krankheiten einsam machen können und welche Krankheiten durch Einsamkeit (mit-)verursacht werden. Manches „Henne-Ei-Problem“ ist dabei noch ungelöst, aber die hohe Relevanz der Einsamkeit für die Gesundheit ist unstrittig. Längsschnittstudien konnten inzwischen auch die kausale Rolle von Einsamkeit für gesundheitliche Beschwerden und Erkrankungen belegen.

Dennoch sind noch viele Fragen offen. Wie ist Einsamkeit eigentlich psychologisch, psychiatrisch zu fassen? Gibt es überhaupt „die Einsamkeit“ als konsistente psychische Entität oder muss man nicht vielmehr von „vielen Einsamkeiten“ sprechen, weil die Einsamkeit eines Menschen, der seinen Partner verloren hat, anders ist als die einer sich unverstanden fühlenden Teenagerin und die wieder anders als die Einsamkeit eines Sterbenden? Wie interagieren proximale und distale Ursachen der Einsamkeit, welche Rolle spielen also persönliche, berufliche oder soziale Aspekte? Welchen Stellenwert hat der Megatrend der Individualisierung in unserer Gesellschaft? Trägt Einsamkeit sogar zur politischen Radikalisierung bei? Und wie laufen die kausalen Pfade zwischen Einsamkeit und Gesundheit? In welche Richtung laufen sie? Für welche augenscheinlich sinnvollen Interventionen zur Verbesserung der Situation von einsamen Menschen gibt es wirklich Evidenz und wie spezifisch müssen sie die unterschiedlichen „Einsamkeiten“ adressieren?

Mehr Fragen als Antworten? Wahrscheinlich ist die aktuelle Situation noch durch viele Fragen und auch viele Antworten geprägt. Das vorliegende Themenheft will beides sammeln, das Forschungsfeld sortieren, neue Studien vorstellen und gesundheitsbezogene Handlungsoptionen aufzeigen. Den Autorinnen und Autoren danken wir für ihre anregenden Beiträge, den Leserinnen und Lesern wünschen wir einen Erkenntnisgewinn, sei es beim Lesen in stiller Einsamkeit oder in der Diskussion der Beiträge im fachlichen Miteinander. Mehr Gemeinsamkeit tut uns in der gegenwärtigen Zeit auf jeden Fall gut.

