# Erratum zu: Einsamkeit: Ein Begriff für viele Wirklichkeiten

**DOI:** 10.1007/s00103-024-03976-5

**Published:** 2024-11-25

**Authors:** Joseph Kuhn, Martin Härter, Peter Brieger, Steffi G. Riedel-Heller

**Affiliations:** 1 Bayerisches Landesamt für Gesundheit und Lebensmittelsicherheit, Veterinärstr. 2, 85764 Oberschleißheim, Deutschland; 2 Zentrum für Psychosoziale Medizin, Institut und Poliklinik für Medizinische Psychologie und Institut für Psychotherapie (IfP), Universitätsklinikum Hamburg-Eppendorf, Martinistr. 52, 20246 Hamburg, Deutschland; 3 Kbo-Isar-Amper-Klinikum, Akademisches Lehrkrankenhaus der LMU, Vockerstr. 72, 85540 Haar, Deutschland; 4 Institut für Sozialmedizin, Arbeitsmedizin und Public Health, Universität Leipzig, Philipp-Rosenthal-Str. 55, 04103 Leipzig, Deutschland

**Erratum zu:**

**Bundesgesundheitsbl 2024**

10.1007/s00103-024-03947-w

Im Originalbeitrag wurden die Korrespondenzautoren unvollständig aufgeführt.

Hier die vollständige Auflistung:
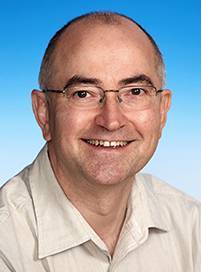


**Dr. Joseph Kuhn**

Bayerisches Landesamt

für Gesundheit und

Lebensmittelsicherheit

Veterinärstr. 2

85764 Oberschleißheim

Deutschland

joseph.kuhn@lgl.bayern.de


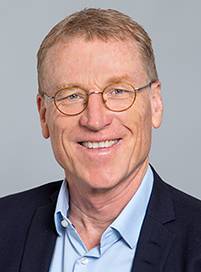


**Prof. Dr. Dr. Martin Härter**

Institut und Poliklinik für

Medizinische Psychologie und

Institut für Psychotherapie (IfP),

Universitätsklinikum Hamburg-Eppendorf

Martinistr. 52

20246 Hamburg

Deutschland

m.haerter@uke.de


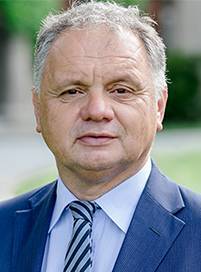


**Prof. Dr. Peter Brieger**

Kbo-Isar-Amper-Klinikum

Vockerstr. 72

85540 Haar

Deutschland

peter.brieger@kbo.de


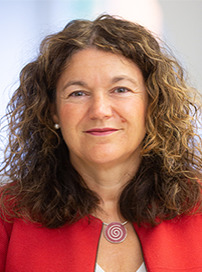


**Prof. Dr. Steffi G. Riedel-Heller, MPH**

Institut für Sozialmedizin, Arbeitsmedizin und Public Health,

Universität Leipzig

Philipp-Rosenthal-Str. 55

04103 Leipzig

Deutschland

Steffi.Riedel-Heller@medizin.uni-leipzig.de

Der Originalbeitrag wurde korrigiert.

